# The significance and expectations of HIV cure research among people living with HIV in Australia

**DOI:** 10.1371/journal.pone.0229733

**Published:** 2020-03-04

**Authors:** Jennifer Power, Gary W. Dowsett, Andrew Westle, Joseph D. Tucker, Sophie Hill, Jeremy Sugarman, Sharon R. Lewin, Graham Brown, Jayne Lucke

**Affiliations:** 1 Australian Research Centre in Sex, Health and Society, La Trobe University, Melbourne, Australia; 2 Centre for Social Research in Health, UNSW Australia, Sydney, Australia; 3 Institute for Global Health and Infectious Diseases, University of North Carolina at Chapel Hill, Chapel Hill, North Carolina, United States of America; 4 Faculty of Infectious and Tropical Diseases, London School of Hygiene and Tropical Medicine, London, United Kingdom; 5 Centre for Health Communication and Participation, School of Psychology and Public Health, La Trobe University, Melbourne, Australia; 6 Berman Institute of Bioethics, Johns Hopkins University, Baltimore, Maryland, United States of America; 7 Department of Medicine, School of Medicine, Johns Hopkins University, Baltimore, Maryland, United States of America; 8 The Peter Doherty Institute for Infection and Immunity, University of Melbourne and Royal Melbourne Hospital, Melbourne, Australia; 9 Department of Infectious Diseases, Alfred Health and Monash University, Melbourne, Australia; 10 School of Public Health, The University of Queensland, Brisbane, Australia; New York University College of Dentistry, UNITED STATES

## Abstract

Most people living with HIV (PLHIV) with reliable access to antiretroviral treatment (ART) have a life expectancy similar to uninfected populations. Despite this, HIV can negatively affect their social and psychological wellbeing. This study aimed to enhance understanding of the expectations PLHIV hold for HIV cure research and the implications this has for HIV cure research trials. We interviewed 20 Australian PLHIV about their expectations for HIV cure research outcomes and the impact a potential cure for HIV may have on their everyday lives. Data were analysed thematically, using both inductive and deductive approaches. The significance of a cure for HIV was expressed by participants as something that would offer relief from their sense of vigilance or uncertainty about their health into the future. A cure was also defined in social terms, as alleviation from worry about potential for onward HIV transmission, concerns for friends and family, and the negative impact of HIV-related stigma. Participants did not consider sustained medication-free viral suppression (or remission) as a cure for HIV because this did not offer certainty in remaining virus free in a way that would alleviate these fears and concerns. A cure was seen as complete elimination of HIV from the body. There is an ethical need to consider the expectations of PLHIV in design of, and recruitment for, HIV cure-related research. The language used to describe HIV cure research should differentiate the long-term aspiration of achieving complete elimination of HIV from the body and possible shorter-term therapeutic advances, such as achieving medication free viral suppression.

## Introduction

Antiretroviral treatment (ART) has significantly reduced morbidity and mortality among people living with HIV (PLHIV) to the point where, with consistent access to and use of ART, they may assume a life expectancy similar to uninfected populations [1] and HIV is now often considered a chronic illness. Furthermore, several large studies provide evidence that there is no risk of sexual transmission of HIV to others from PLHIV whose virus is supressed, summarised by the expression now used widely in HIV prevention, treatment uptake and anti-stigma campaigns, undetectable = untransmittable [2, 3], and pre-exposure prophylaxis medication provides reliable options for prevention of HIV infection [4]. The lower disease burden has lessened the impact of HIV on affected communities with consistent access to ART. However, globally, fewer than half of all PLHIV have reliable access to ART because of major financial, logistical, cultural and political barriers to ART scale-up [5]. In addition, while ART improves immune function, it may not reverse existing negative health effects related to HIV infection. Toxicities associated with long-term ART may also have a negative impact on health [1]. For these reasons, a cure for HIV is still considered clinically and strategically important [1].

In recent years, there has been a growing momentum in scientific research toward finding a cure for HIV infection. This has been supported by the International AIDS Society’s ‘Towards a Cure Initiative’ and other international networks focused on HIV cure research [6]. Globally, there are a number of early stage (phase I/II) clinical trials exploring potential pathways to achieve a cure for HIV. The risks and benefits of being involved in these trials have been well documented, showing that some trials present a risk to the health of PLHIV who participate due to interruption of ART for research purposes [7]. Despite this, recent studies have indicated PLHIV in high-income countries, including the United Kingdom, United States, Thailand and Australia, are likely to be motivated to participate in HIV cure research for altruistic reasons, including a desire to help future generations and/or to advance science [8–11].

A small number of studies have explored the expectations of PLHIV for the longer-term outcomes of HIV cure research [12]. In particular, studies have focused on what PLHIV perceive to be the most desired outcomes associated with an HIV cure. For example, a study of PLHIV in the Netherlands found the most highly desired outcomes of a cure were: alleviating uncertainty about future health problems; reducing the negative social impact of HIV associated with stigma; and no longer being concerned about onward HIV transmission [13]. Similar results have been found in British and Australian studies [9, 14]. These studies highlight complexity in the way ‘HIV cure’ is defined. A cure for HIV has often been used in reference to long-term, medication-free, viral suppression (also called HIV ‘control’ or ‘remission’) [15], as well as a cure in which HIV is eliminated from the body (sometimes referred to as a ‘sterilizing’ cure) [16, 17]. These outcomes could be quite different with respect to whether PLHIV consider themselves to be cured of HIV or not, particularly if they are looking to achieve long-term certainty about their health and inability to transmit HIV, or if a cure is defined as being HIV antibody negative. Understanding the perspectives of PLHIV on HIV cure outcomes sheds light on assumptions about, and desires for, HIV cure research held by populations who are most affected by HIV and who are likely to be involved in future observational and interventional clinical trials [12, 18].

This paper reports findings from a research project in which Australian PLHIV were interviewed about issues likely to influence their willingness to participate in clinical trials related to HIV cure research, including the perceived impact that a cure for HIV would have on their lives. Here, we present findings focused on the personal significance of a potential HIV cure to study participants and the way that this influenced their perceptions of HIV cure research. This analysis follows on from our previous report of findings from this study on willingness to participate in HIV cure trials in Australia [11].

## Materials and method

The study involved one-to-one, in-depth interviews investigating the different ways PLHIV think about a possible cure and how they might consider participation in cure-related trials. Qualitative interviews such as these are highly suited to revealing values, experiences, expectations and ideas explored interactively and conversationally.

### Recruitment

The study inclusion criteria were: living with HIV; aged over 18 years; and living in Australia. The study utilised purposive sampling, whereby participants were recruited through advertising distributed by HIV community organisations, HIV clinics and relevant online social networks. The advertisements invited PLHIV to contact the researchers if they were willing to be interviewed about the social and ethical implications of HIV cure research. Prior to the interview, researchers (AW and JP) had informal conversations with each participant to ensure any questions they had about the study or the researchers could be answered and to get a sense of any potential vulnerabilities or concerns. All participants were provided with information about professional and peer support services in their local area for PLHIV. Ethics approval for this study was granted by the La Trobe University College of Science, Health and Engineering Human Ethics Committee (S15-152).

### Data collection

Twenty PLHIV were interviewed in 2016. Interviews took approximately one hour and were conducted either via telephone or face to face. Interviews were conducted by two of the researchers (AW and JP). Oral consent to participate was obtained from all participants prior to the interview and with a signed consent form. No compensation was provided for participation. Interviewees were informed that the interviewers were researchers who had prior experience working in HIV social research and the study was in no way connected to clinical research toward a cure for HIV.

Interviews followed a semi-structured guide (Table 1) that explored issues or queries that had been raised at a community forum on HIV cure research for PLHIV and PLHIV advocacy groups held in Melbourne, Australia, in February 2016 [19]. The interview guide was designed to explore participants’ general knowledge of, and assumptions about, HIV cure research and their attitudes toward participation in related clinical trials. Interviewers used prompts and probing statements to encourage participants to expand on the topics raised in each question [20]. Participants were also presented with short vignettes (Table 1) that posed hypothetical scenarios relating to participation in HIV cure clinical trials in which the risks and benefits of participation were described. The use of vignettes was intended to encourage participants to reflect on possible points of tension or contradictions in their thinking about the risks and benefits of participation in HIV cure research [21]. In the final section of the interview, participants were asked to explain how they interpreted a set of ‘closed’ questions on willingness to participate in HIV cure research trials (Table 1). The original aim of this was to validate these questions for use for future quantitative survey research. However, we found that participants’ responses to these questions contained rich information on their perceptions of what a cure for HIV would mean for their lives. As such, we included responses to these interview questions in the dataset for the current analysis.

**Table 1:** Interview guide.

General questions	When you were coming in to do this interview today, was there anything you were thinking or wondering about in relation to the topic of the interview that you can share with me? Do you think that a cure for HIV will become available in your lifetime? (Why/why not?) Have you ever deliberately searched for information about HIV cure? (eg. Googled, asked doctors?) Can you tell me more about why you were searching? Can you tell me what you know, if anything, about recent research aimed at developing an HIV cure? Do you recall where you found this information? How do you feel about this recent research? How do you think other PLHIV feel about this research? Are there circumstances in which you would consider participating in a clinical trial relating to HIV cure research? (Why/why not?) What do you imagine this type of clinical trial might involve? Do you imagine such a trial might involve risks to your health? What sort of risks? What level of risk would you be willing to take? What would have to happen for you to consider yourself cured of HIV? How do you feel about the following cure scenarios: You no longer need to take HIV medication, and you cannot pass the virus on to others, but you could potentially get HIV again (you have no natural immunity). You still have HIV, but you do not need to take daily medication to maintain viral suppression. Your viral load is undetectable and you no longer need medication to maintain viral suppression, but doctors are unsure if your HIV will rebound. You will need regular blood tests to monitor viral load for at least the next 5–10 years. If a cure for HIV were to become available, how would it change your life?
Hypothetical scenarios posed	A clinical trial requires you to go off your antiretroviral medication which means your viral load may be unpredictable for up to one year. The trial is unlikely to provide any personal benefit to you but may help develop scientific understanding that could eventually lead to a cure for HIV. Would you be willing to participate in this trial? Why/why not? What, if any, are your major concerns? A clinical trial will involve taking extra medication on top of your antiretroviral medication for a period of two weeks. The impact of this new medication on your viral load is unknown and there may be some side effects. Would you be willing to participate in this trial? Why/why not? What, if any, would be your major concerns?
Interpretation of questions (interviewees were asked to tell us what they perceived to be the main information each question was seeking to elicit)	If you had the opportunity to participate in an HIV cure-related clinical trial beginning tomorrow, how willing would you be to participate? (Not at all willing; somewhat unwilling; somewhat willing; very willing) If you indicated you are willing, to what extent would the following affect your willingness to participate? (Decrease willingness, no effect on willingness, increase willingness) If participation would increase scientific understanding of a potential HIV cure but bring no benefit to your health If participation would help future generations find a cure for HIV but bring no benefit to your own health If participation would allow you to go off medication for a short period and still maintain viral suppression If participation would give you access to specialist HIV medical care as part of the trial If you were guaranteed access to medical care for the rest of your life if participation had a negative effect on your health If participation may result in your viral load increasing or being less predictable for up to one year If participation meant you may develop resistance to your current antiretroviral combination If participation would make you more susceptible to disease and illness If participation would require tissue biopsies or other invasive procedures If participation required weekly visits to a medical clinic for several months

### Data analysis

The interviews were transcribed verbatim and participants had the option of reviewing their transcript to edit or remove any details. They were also able to withdraw their consent to participate after viewing their transcript, although no participants chose to do this. One researcher (AW) verified the transcripts for accuracy and removed identifying details. Two researchers (AW and JP) independently conducted a thematic analysis. Data were initially coded to explore pre-determined themes: perceptions of HIV cure, and willingness to participate in clinical trials [22–24]. Following this, inductive thematic analysis was undertaken to identify emergent themes, organise codes into broader themes, identify sub-themes, and explore complexities or contradictions in the data. Researchers identified core themes by paying attention to emphasis and repetition in the data. To ensure rigour, a process of constant comparison was used, whereby researchers routinely cross-checked data in interviews that had previously been analysed to look for consistency or contradiction with emergent themes [25]. This process was also intended to achieve theoretical saturation of codes. By interrogating each transcript several times, and by re-reading transcripts as the researcher’s ideas or perspective developed in response to subsequent transcripts, each researcher aimed to ensure there were no codes that were missed or ignored [26]. We do not, however, claim that full data saturation was achieved with this analysis given that time and budget constraints did not allow for more interviews to be conducted to further explore themes that had only limited data. Once each researcher had completed an independent analysis of the data, consistencies and discrepancies in the findings of each researcher were identified through discussion and collaboratively writing notes and memos. The coding tree developed through this analysis had broad themes as a base related to participants’ attitudes toward the possibility of a cure for HIV and toward HIV cure research. For example, one broad theme was: “Perceptions and expectations for a cure”. Coding nodes under this included (among others): describing the physical impact of cure, describing the social impact of cure, anticipating life changes that would come from a cure, minimising the impact of a cure, wondering about a cure, hoping for a cure, avoiding cure information, searching for cure information, imagining life without HIV, feeling cynical about a cure, feeling hopeful about a cure, feeling cautious about engaging with cure information, and imaging cure as relief. These nodes were then further refined to unpack nuance and meaning. For example, codes under the node “feeling cynical about a cure” included: fearing disappointment, managing emotions, maintaining a positive attitude toward HIV, mistrusting motives of big-business, and moving between hope and cynicism. The use of gerunds (words such as feeling, moving, wondering, hoping) in line-by-line coding is a technique recommended by Charmaz and Belgrave (2012) to assist researchers to move the analysis away from issues or topics to engage with what participants are doing or feeling [27]. NVivo 12 software was used to organise the data and manage coding, although this was augmented with manual methods, including each researcher reading hardcopies of the transcripts and making notes and highlights prior to NVivo coding. Memo writing was conducted using standard Word software.

### Participant characteristics

Eighteen men and two women were interviewed. The majority of men (n = 16) identified as ‘gay’ or other non-heterosexual identities such as ‘pansexual’ or ‘men who have sex with men’. This is consistent with the pattern of the Australian HIV epidemic where, in 2016, the majority of HIV transmissions were among gay or bisexual men of Anglo-Celtic background [28]. The two women both identified as heterosexual. All participants spoke English fluently, although one indicated that English was his second language. We did not elicit participants’ cultural and ethnic backgrounds.

Participant’s ages ranged from 23 to 64 years and the length of time they had been living with HIV ranged from less than six months to 31 years. Five participants had been living with HIV for three years or less, three of these for less than six months. Ten participants described themselves as closely involved in the HIV community sector, either as employees or volunteers in advocacy and support agencies, or as peer counsellors. Six participants had no contact with the HIV community sector. In lieu of providing a table listing participant characteristics, we have identified the age, gender, sexuality and length of time living with HIV of participants whose interviews have been quoted in the results section of this paper.

## Results

All participants interpreted a cure as being ‘HIV free’, i.e. elimination of HIV from the body. Being asked about how a cure for HIV would change their lives typically led participants to reflect on the impact of HIV on their lives to date as a way of identifying the likely social and psychological benefits of being HIV free. In a separate question, participants were asked about the acceptability of other possible outcomes of cure research such as sustained viral suppression or remission. No participants considered these to be a cure for HIV. Rather, a cure was something that offered certainty that HIV would not return, their viral load would not rebound, and they could no longer transmit HIV to others. Treatments or interventions that offered less certainty were seen as welcome advances in HIV treatment, but not as a cure.

Three key themes emerged relating to the meaning and significance this definition of a cure held for participants: (1) a cure would offer certainty about future health; (2) a cure would mean an absence of everyday worry; and (3) a cure would be relief from the burden of stigma. Participants tended to hold both hope and uncertainty about the potential for a cure to be achieved.

### A cure offers certainty about future health

Participants were asked what physical, emotional or life changes would need to occur for them to consider themselves cured of HIV and what impact this would have on their lives. Health, not surprisingly, was a central theme in most participants’ reflections on the benefits a cure for HIV would bring to their lives. However, participants did not describe this as an improvement in their current state of health. Rather, a cure for HIV was seen to offer freedom from anxiety about future ill health. This was mentioned by participants who had a range of experiences with HIV, including those who were newly diagnosed and had not experienced ill health associated with their infection as well as those living with HIV longer-term.

Well, see, if a cure did become available, oh, it would remove a question I have about my survivability into the future. It would remove questions that I have about very long-term maintenance on antiretroviral therapy … So, I don’t know–well really, it would be about removing the questions about what’s going on in the future.

(Male, gay, 54, living with HIV for 25 years)

[Since] becoming HIV positive, I’ve had this general overarching concern of just getting sick, even though it doesn’t necessarily make much sense, because my viral load is very low and the agency of the HIV is quite–is pretty minimal in the affairs of my health at the moment.

(Male, gay, 23, living with HIV for four months)

Participants were looking for certainty when it came to a cure; a ‘cure’ that did not guarantee permanent viral suppression or elimination was not considered a cure because it did not offer relief from anxiety about future ill health, as one participant stated:

[A cure would mean] that I wouldn’t be worried that what if I got a flu or, you know … if you got shingles, or you know something that’s meant one of the other viruses to go a bit wild, would that set off the HIV again?

(Male, gay, 46, living with HIV for 22 years)

When questioned about their perceptions of sustained medication-free control of HIV (or remission) versus elimination of HIV, participants saw long-term control as an advance on HIV treatment rather than a cure–something that was welcomed and considered important, but without offering the same level of certainty, as the following participants stated:

Remission is not [a cure]. Cure would be knowing it can’t come back, knowing that any reservoir of HIV would not be reactivated if I ceased medication, that it would be undetectable as a HIV test in my body, not just undetectable viral load … I think that’s my stopping point around cure. It would have to be confirmed HIV negativity.

(Male, gay, 52, living with HIV for 22 years)

Do you know what that [idea of remission] makes me feel? Cheated, actually. That’s not a sufficient outcome; it’s a step towards an outcome. You know, I struggle with this as a concept with cancer. They’re in remission. They’re in remission. They’re in remission. And then the cancer’s gone. In a sense, it screams that we’ve put all the time and money and research into only getting to second base. It’s not a home run and we need the home run. We need the home run to push back against the stigma and discrimination, because second base isn’t going to cut it.

(Male, MSM, 43, living with HIV for 18 years)

Living with HIV was expressed by participants as a state of uncertainty about their future. While participants did not describe this as something they held consistently at the front of their minds, questions about what it would feel like to live without HIV evoked responses that described this sense of uncertainty, vulnerability and vigilance about their health. This sense of vulnerability was related to the knowledge of HIV being in their body rather than the physical impact of HIV or of daily ART regimens.

### A cure would mean an absence of everyday worry

Participants offered complex answers to questions about the effects a cure for HIV might have on their lives beyond health. Several participants initially responded by explaining that being cured of HIV would not have a major impact on their everyday lives apart from not having to take medication. Then, as participants thought more about the question, they identified potential social and emotional benefits. The following quotation illustrates this well:

How would [a cure] change my life? It would not change my day-to-day life to be honest because of the way I live. It would–there is an underlying fear and worry with regards to my family. My partner is not HIV positive; neither are any of my children. Thank goodness. So–and I actually haven’t disclosed to any of my children, just because I’ve been well, and it hasn’t been necessary–and as they get older, they’ve got so much to deal with anyway just being children, that we’ve chosen not to tell them. So, in that way, it would give no relief to them. It would be lovely not to have to take medication every day and to just have that underlying, you know, that little-voice worry if I get a cold. It still happens, you know, if something–if I don’t get better within a week, then there’s always that little worry, that niggling little fear. I know that my mother and father would be very thankful, and they would–they’re getting older–and they would stop worrying. But day to day, you know, like, I’m not on any benefits [government financial support], I’m not–I just live quite a normal life. So, day to day, it wouldn’t affect me, but it would have a huge psychological effect on me, I’m sure, and the rest of the people who know me and worry about me.

(Female, heterosexual, 44, living with HIV for 18 years)

As is also evident in the above quotation, one desired outcome of cure for participants was relief from worry. In many participants’ responses, HIV was described as creating a psychological burden relating to anxiety about health, relationships or social interactions, and fear of onward transmission of HIV, which people held despite knowledge of the effectiveness of ART in preventing this.

[With long-term viral suppression] then I could potentially have unprotected sex and not infect anyone, have kids and, like, all of the research that’s gone into that. But, I know it’s [HIV] in my DNA, and I know I’m not cured … For me, cured is that I don’t have to think about it. And the quality of the medicine has almost put us to that point. The last step is the mental.

(Male, gay, 37, living with HIV for four months)

Well, you couldn’t imagine how much [a cure] would change my life. Not being sick isn’t the only thing. It doesn’t erase my past. It doesn’t erase the knowledge of this virus is unlike any other type of illness, and it’s like a cloud hanging over you, your whole life.

(Male, heterosexual, 43, living with HIV for 31 years)

[Being] able to pass on [HIV] is a concern as well, that’s the main fact. I guess, when it comes to my own body, I can live a fairly normal life just taking a pill a day so the biggest thing for me is being able to pass it on to someone else anyway and so yeah–the cure would hopefully mean that I am free of it that I wouldn’t have a risk of infecting someone else–that’s more my own body I guess.

(Male, heterosexual, 45 years, living with HIV for 2 years)

Participants described their view of living free from HIV as relief from tensions or burdens in their social relationships–with their families and partners. These responses are a reminder that living with HIV, is a deeply social experience. The physiological impact is only one aspect. As is evident in the quotations above, HIV that is well managed with ART presents few physical burdens and minimal interruptions to life for many people. The burden of HIV comes from its social impact. As such, the definition of a cure provided by participants was attuned to the social and relational aspects of HIV more than the physical.

### A cure as relief from the burden of stigma

The impact of HIV-related stigma was implicit (and, at times explicit) in most participants’ responses to a question in how a cure for HIV would change their lives. For some, being cured of HIV–which was defined by most participants as no longer being identified as HIV positive–would alleviate the burden of stigma that comes with being seen by others as potentially ‘infectious’, as one participant explained:

[A cure for HIV] would remove that sense of being, as it were, infectious or toxic to other people …You don’t have that kind of stigma. The issue of disclosing that you’re positive, sort of, goes out of the window. And, you know, one is left in the situation where it would have been if you’d never had this illness in the sense of you’ve got to contend with whatever life throws at you in terms of aging and other circumstances, but you no longer have that stigmatized disease.

(Male, gay, 62, living with HIV for 21 years)

Look [a cure] would change my life because I wouldn’t have to be so careful about everything that’s… yeah look, the other thing is I am not open [about HIV status] with people; only close people I tell, you know. It’s that constant fear of people–because there’s a lot of misunderstanding out there in the public … Yeah, and I suppose there’s always a fear that you can get painted with a brush because of that lack of understanding–that probably would be the main thing. I mean outside the obvious not having to take a pill a day or getting worried if you missed one of your medications doses–that’s an inconvenience more than anything else, but yeah, lifestyle, the fear of passing it on–and sexually transmitted or something like that it would be a lot of weight off your shoulders. [Is that something you’re worried about quite often?] Yes, I am … I am married. My wife [and I], we always use condoms, but it would be nice not have to do that, and yeah, the other side of it would be that even immediate family don’t know. If they come and stay over, I am hiding all the meds and things like that. Just those two factors would be… a relief that’s off your shoulders.

(Male, heterosexual, 45, living with HIV for 2 years)

Participants described this burden of stigma in terms similar to the persistent low-level anxiety illustrated above: stigma was experienced as a daily awareness of HIV that demanded some form of emotional management to cope with negative feelings. As one participant said:

I think it’s 25 years that I’ve had HIV. I think I’ve developed lots and lots and lots of compensatory and defensive mechanisms about the fact of living with HIV. And that’s a lot to do with, I don’t know, developing resilience or realising resilience, or dealing with internalised stigma or dealing with the shame from, you know, not keeping myself negative, and I had all the lessons and choices and consequences that I’ve made. And there’s something that I kind of underestimated about that continual low-level burden of maintaining that kind of equilibrium around that sort of stuff. And I don’t know whether cure would ameliorate that or not; but if it did, I think that would be a fine thing. Because, although I work in the sector and stuff, I don’t spend a lot of time talking about this. That’s one of my compensatory mechanisms. But sitting here talking a lot about it. I’m just kind of conscious that it is an all-pervasive kind of burden that, but even if it’s to be ignored it takes up energy to ignore it.

(Male, gay, 54, living with HIV for 20+ years)

As noted previously, the negative impact of living with HIV for many people whose HIV is well managed by ART is social more than physical. This was most apparent when participants spoke about the impact of HIV-related stigma. As can be seen in the quotations above, participants’ responses were powerful illustrations of the ways in which stigma shapes the everyday lives of many PLHIV–some people continually check or modify their emotional responses to internal feelings of shame or stigma, while others are vigilant about what friends and family see or know. Again, these findings suggest that participants’ understanding of a cure incorporates the social aspects of living with HIV.

## Discussion

This paper adds further dimensions to existing research on the expectations for HIV cure research held by PLHIV [13, 29, 30] by exploring the significance and understanding among Australian PLHIV of the potential impact a cure might have on their everyday lives. In particular, these findings explicate how a cure for HIV is thought to improve the lives of PLHIV who are already in good health due to routine access to ART. For participants in this study, living with HIV brought a level of daily anxiety about social and relational complexities, stigma, and sustaining good health into the future. ART did not fully relieve these anxieties, with many participants describing experiences of consistently ‘looking over their shoulders’ to check they were okay. This form of vigilance, particularly about future ill-health or the impact of HIV-related stigma, is an ongoing part of the experience of living with HIV for many people that has been documented in various other studies [31, 32]. Even if such worry is low level, it can be constant and wearing, negatively affecting an individual’s overall quality of life [33, 34]. A similar phenomenon in cancer is referred to by the term ‘fear of recurrence’ [35]. However, one important difference between HIV and cancer is disease transmissibility, which adds a higher level of social complexity and contributes to the stigma PLHIV experience.

The potential for HIV stigma to be relieved by biomedical treatment, including a cure for HIV, has been explored elsewhere [30, 36]. These studies have concluded that HIV-related stigma is complex and layered by other forms of discrimination, including homophobia and discrimination against injecting drug users and sex workers [30]. The way HIV is perceived in the social world is a product of social, political and historical factors, as much as it is the biological nature of the virus. A cure for HIV would obviously be a major factor shaping social understanding and attitudes toward HIV, but it would not be the only factor [37]. ART and viral suppression already mean that HIV is, for those who have access, a manageable chronic condition rather than a terminal illness. It also means HIV is sexually untransmittable (undetectable = untransmittable, U = U) [3]. Despite this, HIV-related stigma endures in ways that have not entirely been changed by ART or U = U [37]. This was evident in this study, as participants described being burdened by fears of onward HIV transmission or negative social reactions. Given this, a biomedical cure is unlikely to mean HIV is no longer a stigmatised condition. However elimination of the virus from the body may relieve the everyday impact of stigma. PLHIV would no longer have to live long-term with the fear, worry and vigilance that was described by participants in this study. Participants expressed uncertainty that sustained virological suppression or remission would be sufficient to achieve this, although it would be welcomed as an important advance on existing treatment. Similar conclusions have been drawn in other studies, which have shown that PLHIV want certainty that the virus will not return before they consider themselves cured, as they mistrust virologic suppression to be sustained long term [9, 38, 39]. In this study, being cured of HIV was defined by participants as being definitively virus free–complete elimination of HIV from the body.

There has been much discussion among researchers working in the HIV cure field about the ethics of using HIV cure terminology to describe research for which a likely outcome is therapeutic interventions to achieve sustained medication-free suppression of HIV (or remission), rather than elimination of HIV from the body [16, 18, 40]. Many PLHIV may be likely to participate in research aimed toward developing therapies for medication-free viral suppression due to the benefits of reduced reliance on everyday ART [8]. However, the aspiration for HIV cure research held by participants in this study was the longer-term goal of being HIV free. There is an ethical need to consider the ways in which HIV cure research is presented and discussed in forums such as media reporting, including the terminology used to describe trials for which elimination of HIV from the body is unlikely to be achieved in the shorter term [15].

Use of specific terminology in public and media reporting on HIV cure research will also help to ensure community expectations for HIV cure research are realistic. This is important to sustain levels of public trust and engagement in HIV science over the longer term. The tension with this, however, is that the aspiration and language of ‘cure’ is powerful and captures attention and imagination that attracts funding and support for research. There is a challenge in maintaining energy and focus on the longer-term goal of achieving elimination of HIV from the body while also ensuring affected communities are presented with realistic expectations of shorter-term research outcomes. Active involvement of both researchers and community advocates in the design and delivery of public messages about HIV cure research will help to ensure messages are created with due consideration to how they may be received by affected communities and PLHIV. In this respect, fulfilling the global principle of ensuring greater and meaningful involvement of PLHIV (GIPA/MIPA) in research is politically and ethically important, but also plays a role in managing quality and accuracy of research output [41].

The findings of this study also suggest there may be value in further research to explore acceptability among PLHIV of therapeutic interventions designed explicitly to achieve sustained, medication-free, viral suppression or remission. If PLHIV are seeking certainty with respect to viral suppression in order to allay anxiety about onward transmission and future ill health, there will need to be a high level of confidence that any new therapeutic interventions will provide this level of certainty.

These findings build on existing research about the reasons why PLHIV might be willing to take risks with their health to volunteer for HIV cure clinical trials. Various studies have shown that PLHIV in many parts of the world have expressed willingness to be involved in HIV cure research for social and altruistic motivations, including a desire to give back to communities that have supported them [8, 11, 42]. Some PLHIV already are involved in early stages of HIV research towards a cure [11, 14, 43]. There is a strong culture of support for research in all sectors of the HIV community in Australia and internationally [44, 45]. This suggests that many PLHIV are conversant with research, its processes, its strengths and limitations, and are familiar with many long-serving researchers in the HIV field. However, HIV cure research is different from other HIV-related research in that it asks PLHIV to take a risk with their current state of good health to be part of early stage research and, potentially, threaten the gains they have achieved in securing that health for no likely clinical benefit. For example, many HIV cure-focused clinical trials involved analytic treatment interruptions, during which participants are required to suspend ART for a period of time, and which may increase the chance of onward HIV transmission through sexual contact–something interviewees in this study were clearly concerned about [7, 46–48]. However, findings from this study show that, despite the effectiveness of modern ART, a cure for HIV would have a significant psychological and social impact on the everyday lives of PLHIV. PLHIV may consider the risks of participation in an HIV cure-related clinical trial through this lens. Even among PLHIV who are physically healthy, there may be vulnerabilities and stressors associated with living with HIV that might reduce people’s perception of their wellbeing when weighing up risks [40]. In addition, living with everyday stressors and vulnerabilities associated with HIV may be what makes participation in research toward a possible cure meaningful and significant for PLHIV in ways that are difficult to assess objectively [42]. Again, this speaks to the importance of including PLHIV on clinical trial research teams so perspectives gained through the experience of living with HIV can inform these trials.

### Limitations

There are limitations to this study to consider. First, being located in Australia, all participants in this study had access to publicly funded healthcare and subsidised ART. This may not the case in many other settings. The relevance of a cure for HIV, and the significance of sustained, medication-free, viral suppression, might be different for PLHIV who have no or less reliable access to ART. The sample size was also small and lacked ethnic or cultural diversity, so should not be considered reflective of the perspectives of all PLHIV.

## Conclusion

Despite HIV being a manageable condition for people with access to ART, a cure that eliminates HIV from the body would fundamentally change the lives of many PLHIV. As shown in this study, the impact of HIV affects an individual’s social, psychological and emotional life, extending far beyond physical health alone. The cure most desired by PLHIV is one that can relieve or reverse these negative impacts [40]. This is important to consider in the context of the ethics of HIV cure research. Research that considers the lived experiences and perspectives of PLHIV is necessary to determine acceptability of HIV cure research and trial participation as well as likely outcomes from such research. The hopes and expectations that PLHIV and affected communities hold for HIV cure research may not align with the possible shorter-term outcomes of current cure research. This is so because the more likely shorter-term outcome from research toward a cure for HIV is sustained, medication-free, viral suppression (or remission), which, while significant, is very likely to be considered by many PLHIV as an extension of treatment rather than a cure [40].
